# Platelet and Red Blood Cell Volume Indices in Patients with Rheumatoid Arthritis: A Systematic Review and Meta-Analysis

**DOI:** 10.3390/diagnostics12112633

**Published:** 2022-10-30

**Authors:** Angelo Zinellu, Arduino A. Mangoni

**Affiliations:** 1 Department of Biomedical Sciences, University of Sassari, 07100 Sassari, Italy; 2 Discipline of Clinical Pharmacology, College of Medicine and Public Health, Flinders University, Sturt Road, Bedford Park, SA 5042, Australia; 3 Department of Clinical Pharmacology, Flinders Medical Centre, Southern Adelaide Local Health Network, Flinders Drive, Bedford Park, SA 5042, Australia

**Keywords:** mean platelet volume, platelet distribution width, red blood cell width, rheumatoid arthritis, biomarkers

## Abstract

Alterations in the volume of platelets (mean platelet volume, MPV; platelet distribution width, PDW) and erythrocytes (red blood cell distribution width, RDW) have been reported in rheumatoid arthritis (RA) and might serve as diagnostic biomarkers. We conducted a systematic review and meta-analysis of the MPV, PDW, and RDW in RA patients and healthy controls. Relevant articles were searched in PubMed, Web of Science, Scopus, and Google Scholar from inception to June 2022. Risk of bias was assessed using the Joanna Briggs Institute Critical Appraisal Checklist and certainty of evidence was assessed using GRADE. In 23 studies (2194 RA patients and 1565 healthy controls), the RDW, but not MPV or PDW, was significantly higher in RA patients (standardized mean difference, SMD = 0.96, 95% CI 0.78 to 1.15, *p* < 0.001; moderate certainty of evidence). The substantial heterogeneity observed (I^2^ = 75.1%, *p* < 0.001) was virtually removed in a subgroup of prospective studies. In sensitivity analysis, the magnitude of the effect size was not substantially modified by sequentially removing individual studies. There was no significant publication bias. No significant associations were observed between the effect size and pre-defined study or patient characteristics. The results of our study suggest that the RDW might be a useful biomarker for the diagnosis of RA, and complement the clinical information provided by other patient characteristics and laboratory parameters (PROSPERO registration number: CRD42022349432).

## 1. Introduction

The population prevalence of rheumatoid arthritis (RA), the most common form of inflammatory polyarthritis, is up to 1% [1,2]. While the presence of overt clinical manifestations generally leads to a straightforward diagnosis and classification a significant number of patients with RA present with mild, non-specific signs and symptoms [3]. Given the established short- and long-term benefits of early recognition and pharmacological intervention, there is an ongoing search for novel biomarkers that might facilitate the diagnosis of RA, particularly in the early stages [4,5,6]. A number of biomarkers that reflect the presence of an inflammatory state, e.g., rheumatoid factor (RF), anti-cyclic citrullinated protein antibody (ACPA), C-reactive protein (CRP), and erythrocyte sedimentation rate (ESR), are currently in use for the diagnosis of RA [4,5,6]. However, their diagnostic accuracy has limitations. For example, the reported disease sensitivity of the RF, ACPA, CRP, and ESR is 60–90% [7,8], 55–80% [9], 63% [10], and 55% [11], respectively. In terms of specificity, the RF can also be detected in healthy individuals and in patients with other autoimmune conditions. Similarly, elevations in CRP and ESR values are also common in patients with infections and those suffering from other autoimmune and inflammatory states [4,5,6].

To address this important issue, an increasing number of studies have investigated other potential biomarkers for the diagnosis of RA, particularly those that could be accessed or derived from routine laboratory investigations, with potential advantages in terms of sensitivity and/or specificity over available biomarkers. Recent systematic reviews and meta-analyses have highlighted the potential diagnostic role of routinely derived haematological indices, e.g., the neutrophil-to-lymphocyte ratio and the platelet-to-lymphocyte ratio, in RA [12]. Additionally, alterations in the volume and volume distribution of two key blood cell types, i.e., platelets and red blood cells, are known to reflect the presence of a pro-inflammatory state and oxidative stress [13,14,15,16,17]. In this context, routinely derived indices such as the mean platelet volume (MPV), the platelet distribution width (PDW, calculated using the following formulas: standard deviation (SD)/MPV (fL); (SD/MPV) × 100 (%)), and the red blood cell distribution width (RDW, a measure of anisocytosis derived from the following formula: (SD/mean corpuscular volume) × 100 (%)) have also been increasingly investigated as potential inexpensive biomarkers in patients with RA. Therefore, we sought to critically appraise the association between the MPV, PDW, and RDW and RA by conducting a systematic review and meta-analysis of cross-sectional studies comparing these haematological parameters between patients with RA and healthy controls and investigating potential associations between the effect size and several study and patient characteristics.

## 2. Materials and Methods

### 2.1. Search Strategy and Study Selection

We systematically searched the electronic databases PubMed, Web of Science, Scopus, and Google Scholar, from inception to June 2022, using the following terms and their combination: “RDW” or “red cell distribution width” or “MPV” or “mean platelet volume” or “PDW” or “platelet distribution width” and “rheumatoid arthritis”. Two investigators independently screened each abstract. If relevant, they independently reviewed the full article. Eligibility criteria were (i) assessment of MPV, RDW or PDW in plasma or serum; (ii) comparison of MPV, RDW or PDW values between patients with RA and healthy subjects (case–control design); (iii) ≥10 patients with RA; (iv) English language used; and (v) full text available. The references of retrieved articles were also searched for additional studies. Any disagreement between reviewers was resolved by a third investigator. The following data were extracted from each study: year of publication, country where the study was conducted, study design (prospective or retrospective), sample size, age, sex, disease activity score-28 (DAS28), and MPV, RDW, PDW, CRP, and ESR values. The risk of bias was assessed using the Joanna Briggs Institute (JBI) Critical Appraisal Checklist for analytical studies. Studies addressing ≥75% of the checklist items were considering as having a low risk of bias [18]. The certainty of evidence was assessed using the Grades of Recommendation, Assessment, Development and Evaluation (GRADE) Working Group system [19]. The study complied with the Preferred Reporting Items for Systematic reviews and Meta-Analyses (PRISMA) 2020 statement (Supplementary Tables S1 and S2) [20]. The protocol was registered in the International Prospective Register of Systematic Reviews (PROSPERO, CRD42022349432).

### 2.2. Statistical Analysis

Standardized mean differences (SMDs) and 95% confidence intervals (CIs) were calculated to generate forest plots of MPV, PDW, and RDW values in RA patients and healthy subjects (significance level at *p* < 0.05). When necessary, means and standard deviations were extrapolated from medians and ranges [21], or interquartile ranges [22]. The Q-statistic was used to assess between-study heterogeneity in SMDs (significance level at *p* < 0.10). A random-effect model based on the inverse-variance method was used in presence of moderate-substantial heterogeneity, as indicated by I^2^ values of ≥30% [23]. Sensitivity analysis was conducted to investigate the influence of individual studies on the overall risk estimate [24]. The Begg’s and Egger’s tests (significance level at *p* < 0.05) and the Duval and Tweedie “trim-and-fill” procedure were used to assess publication bias [25,26,27]. Subgroup and univariate meta-regression analyses were conducted to investigate associations between the effect size and the following parameters: age, sex, publication year, sample size, study design, country where the study was conducted, CRP, and ESR. Statistical analyses were performed using Stata 14 (Stata Corp., College Station, TX, USA).

## 3. Results

### 3.1. Systematic Search

**Figure 1:**
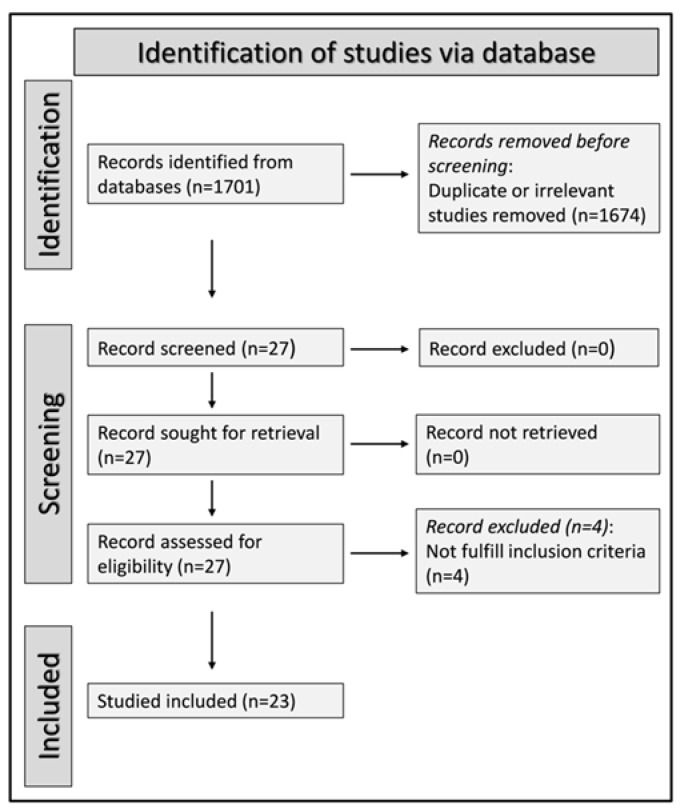
PRISMA 2020 flow diagram.

We initially identified 1701 studies. A total of 1674 were excluded after the first screening because they were either duplicates or irrelevant. Following a full-text revision of the remaining 27 articles, four were further excluded because they did not have a healthy control group. Thus, 23 studies were included in the final analysis (Figure 1) [28,29,30,31,32,33,34,35,36,37,38,39,40,41,42,43,44,45,46,47,48,49,50]. There was no disagreement between the two independent investigators. In all studies, patients with RA had no history of other conditions potentially associated with alterations in MPV, PDW, or RDW values. Data on DAS28 were reported in only three studies [32,34,35]. The characteristics of the retrieved studies, published between 2008 and 2022, are presented in Table 1.

### 3.2. Mean Platelet Volume (MPV)

#### 3.2.1. Study Characteristics

Seventeen studies in 1489 RA patients (mean age 53 years, 77% females) and 1082 healthy controls (mean age 54 years, 71% females) reported data on the MPV [28,29,30,31,32,33,34,35,37,39,41,42,43,45,46,47,50]. Eleven studies were performed in Turkey [28,30,31,32,33,34,35,39,41,45,47], two in Egypt [46,50], and one in Romania [29], India [37], China [42], and Iran [43], respectively. Eleven studies were prospective [29,30,32,34,37,41,43,45,46,47,50] and six retrospective (Table 1) [28,31,33,35,39,42].

#### 3.2.2. Risk of Bias

The risk of bias was considered low in all studies (Table 2) [28,29,30,31,32,33,34,35,37,39,41,42,43,45,46,47,50].

#### 3.2.3. Results of Individual Studies and Syntheses

**Figure 2:**
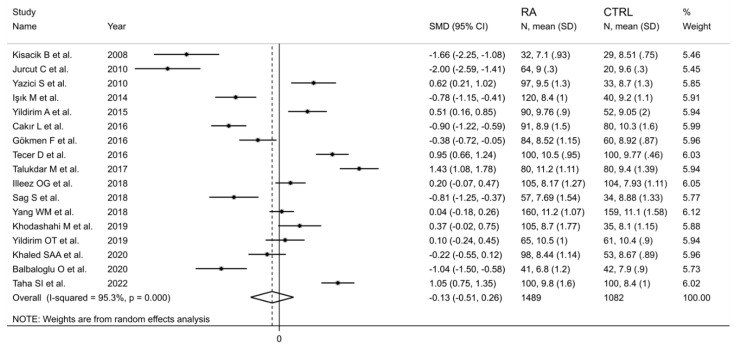
Forest plot of studies examining the mean platelet volume in patients with rheumatoid arthritis and healthy controls.

**Figure 3:**
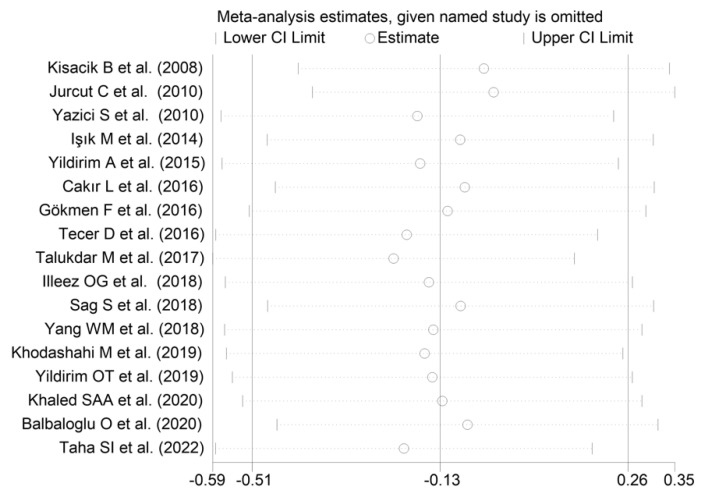
Sensitivity analysis of the association between the mean platelet volume and rheumatoid arthritis. For each study, the displayed effect size (hollow circles) corresponds to an overall effect size computed from a meta-analysis excluding that study.

The forest plot for MPV values in RA patients and control subjects is reported in Figure 2. Given the substantial between-study heterogeneity observed (I^2^ = 95.3%, *p* < 0.001), random-effects models were used. Pooled results showed that the MPV values were not significantly different between RA patients and healthy controls (SMD = −0.13, 95% CI −0.51 to 0.26, *p* = 0.515). In sensitivity analysis, the corresponding pooled SMD values were not substantially altered when each study was sequentially omitted (effect size range, between −0.22 and −0.02; Figure 3).

#### 3.2.4. Publication Bias

**Figure 4:**
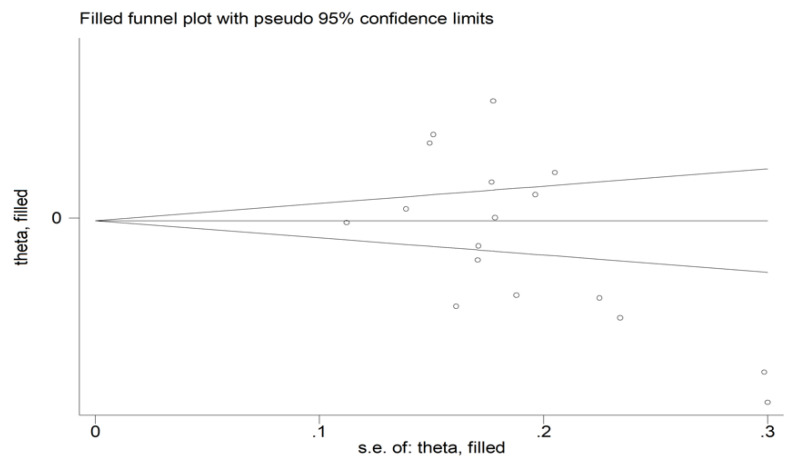
Funnel plot of studies investigating the association between the mean platelet volume and rheumatoid arthritis. Genuine studies are represented by free circles, respectively.

There was no significant publication bias (Begg’s test, *p* = 0.091; Egger’s test, *p* = 0.051). Similarly, the “trim-and-fill” method did not find any missing study to be added to the funnel plot (Figure 4).

#### 3.2.5. Subgroup and Meta-Regression Analysis

**Figure 5:**
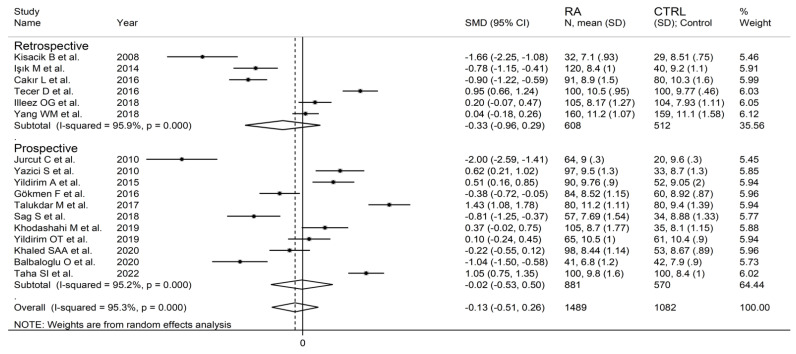
Forest plot of studies examining the mean platelet volume in patients with rheumatoid arthritis and healthy controls according to study design.

**Figure 6:**
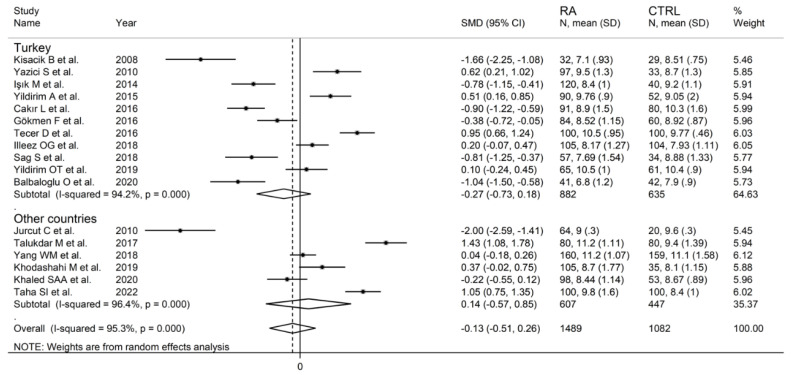
Forest plot of studies examining the mean platelet volume in patients with rheumatoid arthritis and healthy controls according to the country where the study was conducted.

**Figure 7:**
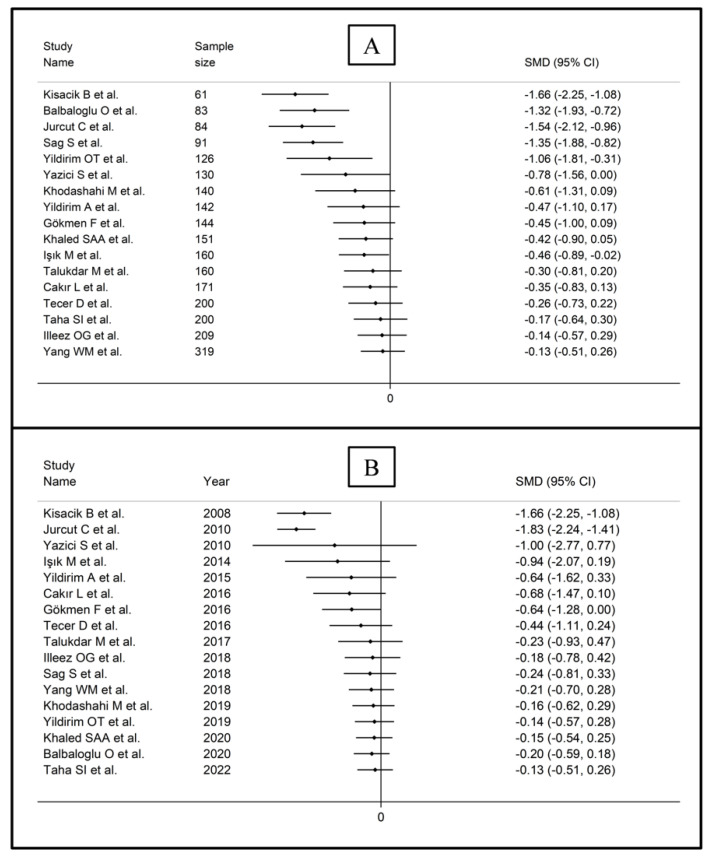
Cumulative meta-analysis of the mean platelet volume based on study design (**A**) and publication year (**B**).

In subgroup analysis, there were no significant differences (*p* = 0.522) in SMD between retrospective (SMD = −0.33, 95% CI −0.96 to 0.29, *p* = 0.30; I^2^ = 95.9%, *p* < 0.001) and prospective studies (SMD = −0.02, 95% CI −0.53 to 0.50, *p* = 0.95; I^2^ = 95.2%, *p* < 0.001; Figure 5). Similarly, no significant differences (*p* = 0.405) were observed between studies conducted in Turkey (SMD = −0.27, 95% CI −0.73 to 0.18, *p* = 0.297; I^2^ = 94.2%, *p* < 0.001) and other countries (SMD = 0.14, 95% CI −0.17 to 0.85, *p* = 0.23; I^2^ = 96.4%, *p* < 0.001; Figure 6). In univariate meta-regression, no significant associations were observed between the effect size and age (t = 1.33, *p* = 0.20), proportion of males (t = −0.89, *p* = 0.39), CRP (t = −0.83, *p* = 0.44), or ESR (t = −1.12, *p* = 0.30). Conversely, there was a significant association between the SMD and sample size (t = 2.30, *p* = 0.036) and a trend toward a significant relationship between the SMD and publication year (t = 1.86, *p* = 0.08), as also evident in a cumulative analysis based on sample size and publication year (Figure 7).

#### 3.2.6. Certainty of Evidence

The initial level of certainty was considered low because of the cross-sectional nature of the studies (rating 2, ⊕⊕⊝⊝). After taking into account the low risk of bias in all studies (no rating change), the substantial and unexplained heterogeneity (downgrade one level), the lack of indirectness (no rating change required), the relatively high imprecision (confidence intervals with threshold crossing, downgrade one level), the relatively small effect size (SMD = −0.13, downgrade one level) [51], and the absence of publication bias (no rating change), the overall level of certainty was downgraded to extremely low (rating 0, ⊝⊝⊝⊝).

### 3.3. Platelet Distribution Width (PDW)

#### 3.3.1. Study Characteristics

Seven studies with in 599 RA patients (mean age 52 years, 74% females) and 364 healthy controls (mean age 50 years, 68% females) investigated the PDW [29,31,32,39,41,45,46]. Five studies were conducted in Turkey [31,32,39,41,45], one in Romania [29], and one in Egypt [46]. Five studies were prospective [29,32,41,45,46], and the remaining two retrospective (Table 1) [31,39].

#### 3.3.2. Publication Bias

The risk of bias was considered low in all studies (Table 2) [29,31,32,39,41,45,46].

#### 3.3.3. Results of Individual Studies and Syntheses

**Figure 8:**
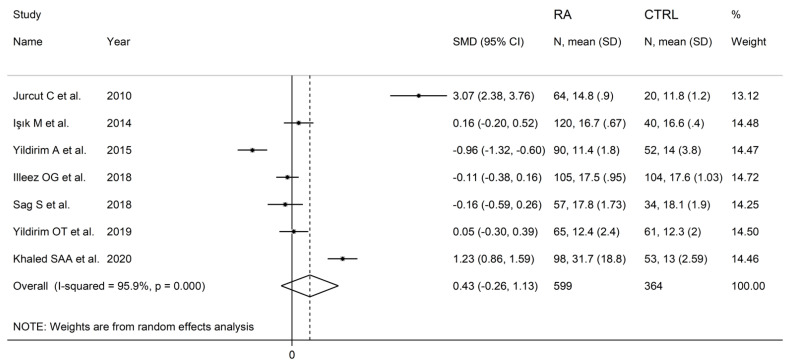
Forest plot of studies examining the platelet distribution width in patients with rheumatoid arthritis and healthy controls.

**Figure 9:**
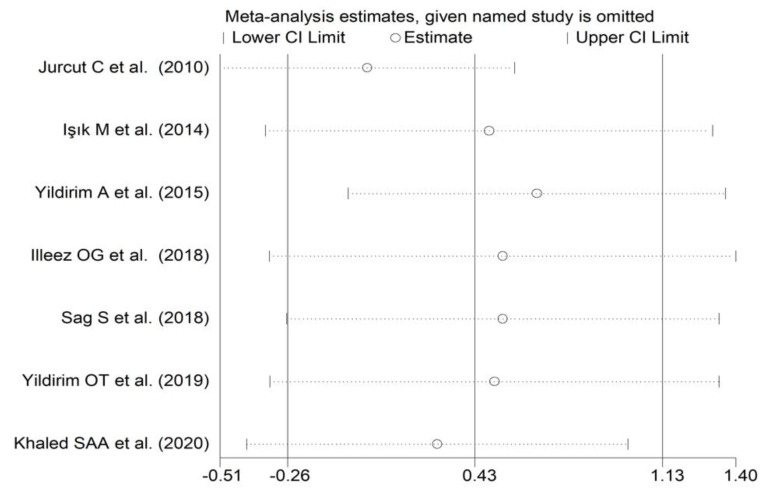
Sensitivity analysis of the association between the platelet distribution width and rheumatoid arthritis. For each study, the displayed effect size (hollow circles) corresponds to an overall effect size computed from a meta-analysis excluding that study.

The forest plot for PDW values in RA patients and control subjects is reported in Figure 8. Random-effects models were used because of the extreme heterogeneity observed (I^2^ = 95.9%, *p* < 0.001). Pooled results showed that the PDW values were not significantly different between the two groups (SMD = 0.43, 95% CI −0.26 to 1.13, *p* = 0.222). In sensitivity analysis, the corresponding pooled SMD was not substantially altered when each study was in turn omitted (effect size range, between 0.03 and 0.66, Figure 9).

#### 3.3.4. Publication Bias

Assessment of publication bias was not possible because of the relatively small number of studies identified.

#### 3.3.5. Subgroup and Meta-Regression Analysis

**Figure 10:**
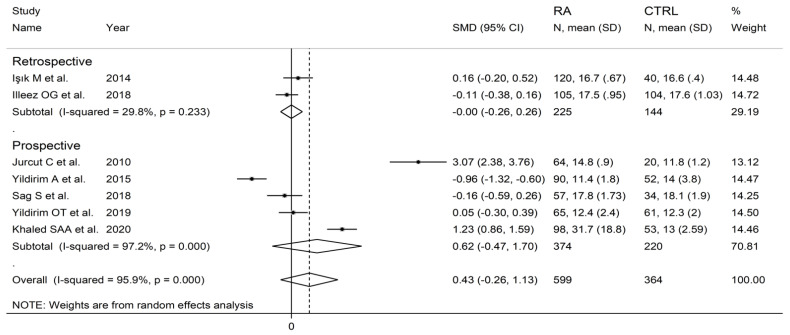
Forest plot of studies examining the platelet distribution width in patients with rheumatoid arthritis and healthy controls according to study design.

**Figure 11:**
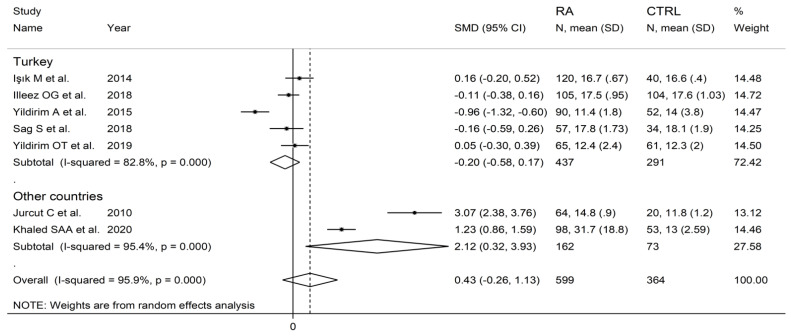
Forest plot of studies examining the platelet distribution width in patients with rheumatoid arthritis and healthy controls according to the country where the study was conducted.

In subgroup analysis, no significant differences (*p* = 0.63) in SMD were observed between retrospective (SMD = 0.00, 95% CI −0.26 to 0.26, *p* = 0.999; I^2^ = 29.8%, *p* = 0.233) and prospective studies (SMD = 0.62, 95% CI −0.47 to 1.70, *p* = 0.264; I^2^ = 97.2%, *p* < 0.001; Figure 10). By contrast, there were significant differences (*p* = 0.012) in SMD between studies conducted in Turkey (SMD = −0.20, 95% CI −0.58 to 0.17, *p* = 0.289; I^2^ = 82.8%, *p* = 0.007) and other countries (SMD = 2.12, 95% CI 0.32 to 3.93, *p* = 0.021; I^2^ = 95.4%, *p* < 0.001; Figure 11). Meta-regression analysis was not possible because of the relatively small number of studies identified.

#### 3.3.6. Certainty of Evidence

The initial level of certainty was considered low because of the cross-sectional nature of the studies (rating 2, ⊕⊕⊝⊝). After taking into account the low risk of bias in all studies (no rating change), the substantial and unexplained heterogeneity (downgrade one level), the lack of indirectness (no rating change required), the relatively high imprecision (confidence intervals with threshold crossing, downgrade one level), the relatively small effect size (SMD = 0.43, downgrade one level) [51], and the absence of publication bias (no rating change), the overall level of certainty was downgraded to extremely low (rating 0, ⊝⊝⊝⊝).

### 3.4. Red Blood Cell Distribution Width (RDW)

#### 3.4.1. Study Characteristics

Eleven studies in 1221 RA patients (mean age 55 years, 80% females) and 983 healthy controls (mean age 53 years, 73% females) reported data on RDW [33,35,36,38,40,42,44,45,48,49,50]. Four studies were conducted in Turkey [33,35,44,45], four in China [36,40,42,49], and one in Egypt [50], Iraq [38], and Bosnia and Erzegovina [48], respectively. Six studies were retrospective [33,35,36,40,42,44], and the remaining five were prospective (Table 1) [38,45,48,49,50].

#### 3.4.2. Risk of Bias

The risk of bias was considered low in all studies (Table 2) [33,35,36,38,40,42,44,45,48,49,50].

#### 3.4.3. Results of Individual Studies and Syntheses

**Figure 12:**
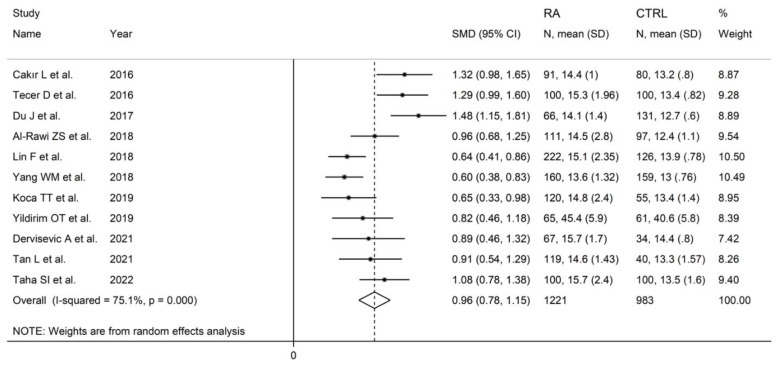
Forest plot of studies examining the red cell distribution width in patients with rheumatoid arthritis and healthy controls.

**Figure 13:**
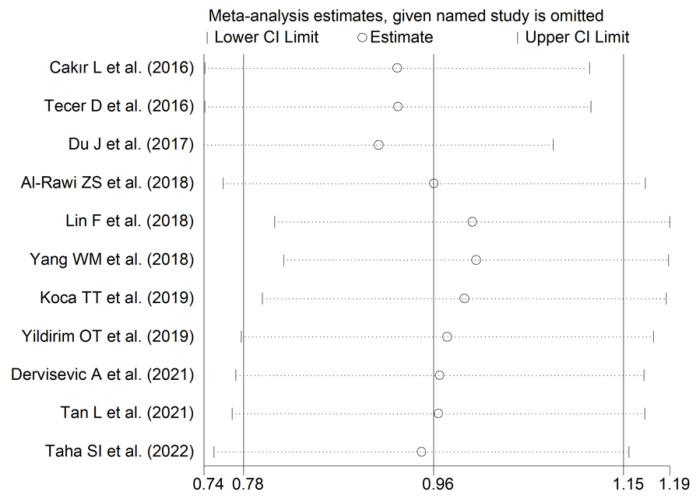
Sensitivity analysis of the association between the red cell distribution width and rheumatoid arthritis. For each study, the displayed effect size (hollow circles) corresponds to an overall effect size computed from a meta-analysis excluding that study.

The forest plot for RDW values in RA patients and control subjects is reported in Figure 12. In all studies, patients with RA had significantly higher RDW values than controls (mean difference range, 0.60 to 1.48). Substantial heterogeneity between studies was observed (I^2^ = 75.1%, *p* < 0.001); therefore, random-effects models were used. Pooled results showed that the RDW values were significantly higher in RA patients (SMD = 0.96, 95% CI 0.78 to 1.15, *p* < 0.001). Sensitivity analysis showed that the corresponding pooled SMD was not substantially affected by sequentially removing individual studies (effect size range, between 0.91 and 1.00; Figure 13).

#### 3.4.4. Publication Bias

**Figure 14:**
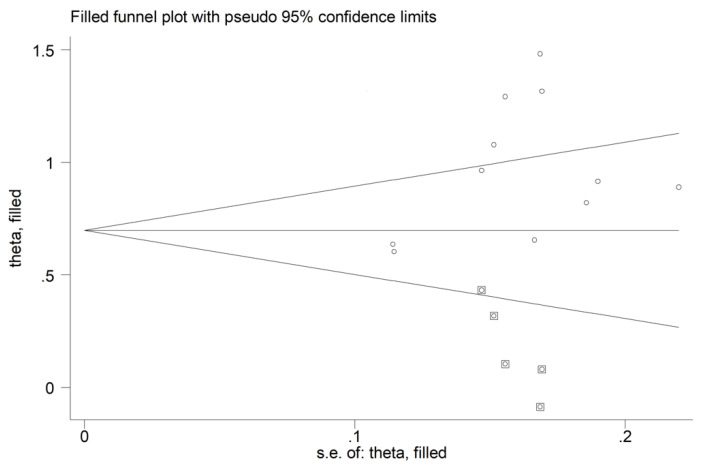
Funnel plot of studies investigating the association between the red cell distribution width and rheumatoid arthritis after trimming and filling. Dummy studies and genuine studies are represented by enclosed circles and free circles, respectively.

There was no significant publication bias (Begg’s test, *p* = 0.44; Egger’s test, *p* = 0.12). The “trim-and-fill” method identified five missing studies to be added to the left side of the funnel plot to ensure symmetry (Figure 14). However, the resulting effect size remained significantly different (SMD = 0.72, 95% CI 0.50 to 0.93, *p* < 0.001).

#### 3.4.5. Subgroup and Meta-Regression Analysis

**Figure 15:**
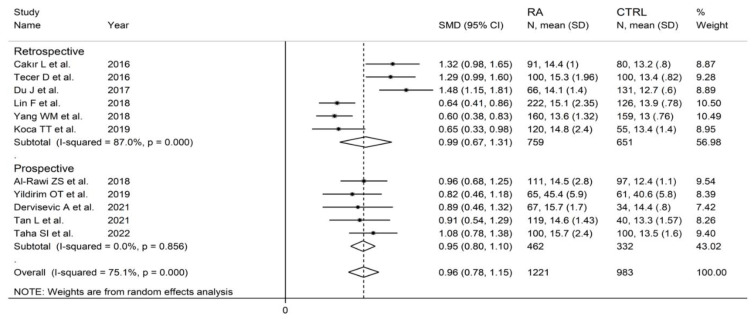
Forest plot of studies examining the red cell distribution width in patients with rheumatoid arthritis and healthy controls according to study design.

**Figure 16:**
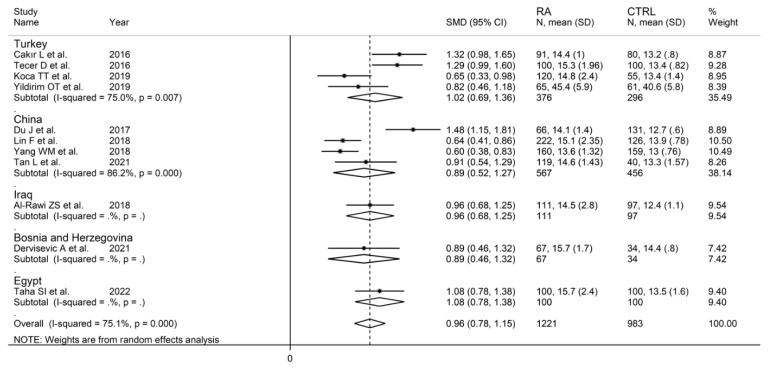
Forest plot of studies examining the red cell distribution width in patients with rheumatoid arthritis and healthy controls according to the country where the study was conducted.

In subgroup analysis, no significant differences (*p* = 0.84) were observed in SMD between retrospective (SMD = 0.99, 95% CI 0.67 to 1.31, *p* < 0.001; I^2^ = 87.0%, *p* < 0.001) and prospective studies (SMD = 0.95, 95% CI 0.80 to 1.10, *p* < 0.001; I^2^ = 0.0%, *p* = 0.856; Figure 15). However, heterogeneity was virtually abolished in prospective studies. No significant differences in SMD (*p* = 0.633) were also observed between studies conducted in Turkey (SMD = 1.02, 95% CI 0.69 to 1.36, *p* < 0.001; I^2^ = 75.0%, *p* = 0.007) and other countries (SMD = 0.89, 95% CI 0.52 to 1.27, *p* < 0.001; I^2^ = 86.2%, *p* < 0.001, Figure 16). In univariate meta-regression, no significant associations were observed between the effect size and age (t = −0.564, *p* = 0.59), proportion of males (t = −1.03, *p* = 0.33), publication year (t = −1.11, *p* = 0.30), or sample size (t = −1.37, *p* = 0.21). Associations between effect size and CRP or ESR could not be performed due to the small number of studies reporting these parameters.

#### 3.4.6. Certainty of Evidence

The initial level of certainty was considered low because of the cross-sectional nature of the studies (rating 2, ⊕⊕⊝⊝). After taking into account the low risk of bias in all studies (no rating change), the substantial heterogeneity that was at least partially explained by the type of study design (no rating change), the lack of indirectness (no rating change required), the relatively low imprecision (confidence intervals without threshold crossing, no rating change required), the relatively large effect size (SMD = 0.96, upgrade one level), and the absence of publication bias (no rating change), the overall level of certainty was upgraded to moderate (rating 3, ⊕⊕⊕⊝).

## 4. Discussion

In our systematic review and meta-analysis, measures of red blood cell volume distribution (RDW), but not of platelet volume and distribution (MPV and PDW), were significantly associated with the presence of RA. In sensitivity analysis, the effect size, SMD of RDW, was not substantially altered after sequentially removing individual studies. In subgroup and meta-regression analysis, the effect size was not significantly associated with pre-defined study and patient characteristics although no formal analysis could be performed with the CRP or the ESR because of the limited number of studies reporting these inflammatory markers.

The distribution of the volume of circulating red blood cells, normally between 80–100 fL, can significantly increase (anisocytosis) in several physiological and pathophysiological states and is expressed as a relative increase in RDW values [16,52,53,54]. Generally, the presence of anisocytosis is favoured by a reduced production and release of mature red blood cells from the bone marrow and/or a reduced destruction of old blood cells in the liver and/or spleen [16,55]. Given that the RA patients recruited in the articles identified in our study did not have any evidence of anaemia secondary to iron, vitamin B_12_, or folic acid deficiency, common causes of impaired red blood cell production and anisocytosis [16,55], it is possible that RA-specific factors might affect erythropoiesis. Samson et al. documented the presence of ineffective erythropoiesis, possibly due to a state of functional iron deficiency or other unknown mechanisms, in a subset of patients with RA [56]. Similar observations have been reported by other authors [57]. This condition seems to differ from the anaemia of chronic disease observed in a substantial proportion of patients with RA [58]. Further studies have reported that the serum concentrations of key pro-inflammatory cytokines, e.g., interleukin 1, are significantly higher in RA patients with anaemia compared to those without anaemia. In further experiments, interleukin 1 was able to significantly suppress the formation of erythroid, but not granulocyte-macrophage, progenitor cells in bone marrow cultures [59]. Notably, a similar inhibitory effect of erythroid progenitor cells has also been observed with serum of RA patients with anaemia but not with serum of non-anaemic RA patients, suggesting the presence of factors other than interleukin 1 in suppressing erythropoiesis in RA [60]. As a consequence of the impaired maturation of erythroid cells in the bone marrow, there is an increased release of immature erythrocytes into the circulation which, in turn, increases the heterogeneity of the volume of red blood cells and the RDW [61]. Looking at the impaired clearance of red blood cells as an alternative mechanism accounting for the higher RDW values observed in RA, increasing evidence suggests a key role of the liver parenchyma in modulating this process through macrophages [62]. Epidemiological studies have reported a significantly higher incidence of liver abnormalities in RA patients compared to the general population, particularly any liver disease (adjusted hazard ratio, aHR, 1.49, 95% CI 1.26 to 1.76), and cirrhosis (aHR 2.07, 95% CI 1.50 to 2.86) [63]. However, whether specific liver abnormalities affect macrophage function and red blood cell clearance requires additional studies. Furthermore, the absence of clinically overt manifestations of liver disease in RA patients in the selected studies suggests that any possible role of an impaired liver-mediated erythrocyte clearance in the reported increased RDW in this group likely involves subtle yet unknown molecular and biochemical mechanisms.

Whilst more research is warranted to investigate the pathophysiological mechanisms underpinning anisocytosis in RA, adequately designed prospective studies are also needed to confirm our findings and to robustly characterize the diagnostic performance of the RDW in RA, singly or in combination with other inflammatory markers and/or clinical and demographic characteristics, by assessing the receiver operating characteristics curve and adequate cut-off values for optimal sensitivity and specificity. In this context, an important observation in our subgroup and meta-regression analysis was the absence of significant associations between the effect size, expressed as the SMD in RDW values between RA patients and healthy controls, and pre-defined study and patient characteristics. Whilst this suggests that the information provided by the RDW might complement, rather than duplicate, that provided from established diagnostic parameters it is also important to emphasise that the lack of relevant data in the selected studies prevented the formal assessment of specific associations between the RDW and the DAS28, CRP, and ESR. Therefore, future studies should ideally assess the diagnostic performance of the RDW and the possible gain, in terms of discrimination, specificity, and sensitivity, compared to established criteria, including biomarkers of inflammation. Only then, can the potential use of the RDW for the diagnosis of RA in routine clinical practice be adequately determined and justified.

Another potential clinical application of the RDW in RA involves its role in predicting outcomes as well as treatment response in this group. In a retrospective analysis of 160 patients with RA, Rodríguez-Carrio et al. reported that the RDW at the time of disease onset independently predicted a cardiovascular event, a common occurrence in RA patients, after adjusting for age, sex, and RA duration (HR 1.25, 95% CI 1.08 to 1.44, *p* = 0.003) [64]. More recently, in 82 RA patients, Bellan et al. reported that the baseline RDW independently predicted treatment response with methotrexate at three months (HR 1.53, 95% CI 1.01 to 2.31, *p* = 0.04) after adjusting for age, sex, haemoglobin, and daily dose of prednisone [65]. Pending the confirmation of these findings in other studies, the RDW might represent a robust, easily derived and relatively inexpensive biomarker with combined diagnostic and prognostic capacity in patients with RA, with significant advantages over existing biomarkers.

The strengths of our systematic review and meta-analysis include the robust assessment of the certainty of evidence using GRADE and the conduct of pre-defined subgroup and meta-regression analyses to investigate associations between the effect size and other study and patient characteristics as well as potential sources of heterogeneity. One potential limitation is the relative lack of studies in European, American, and African cohorts which limits the generalisability of the results. However, the lack of significant differences, in subgroup analysis, in effect size according to the country where the study was conducted suggests that ethnicity does not substantially affect the association between the RDW and RA. An additional limitation is the substantial heterogeneity observed in our analyses. However, the heterogeneity of studies investigating the RDW was virtually eliminated in a subgroup of prospective studies. Furthermore, in the sensitivity analysis, the sequential omission of individual studies did not have a tangible effect on the resulting SMD values.

## 5. Conclusions

The RDW, but not the MPV or the PDW, can significantly discriminate between RA patients and healthy controls. Although this routinely derived biomarker of anisocytosis may enhance the diagnosis of RA, particularly in those patients without overt clinical manifestations of the disease, adequately designed prospective studies are warranted to justify its routine use as a biomarker of RA.

## Figures and Tables

**Table 1:** Study characteristics.

		Healthy Controls			Patients with Rheumatoid Arthritis				
First Author, Year, Country [Ref]	Study Design	n	Age (Years) M/F	MPV (fL) RDW (%) PDW (%)	n	Age (Years) M/F	MPV (fL) RDW (%) PDW (%)	CRP (mg/dL)	ESR (mm/h)
Kisacik B, 2008, Turkey [28]	R	29	5/24	8.5 ± 0.7 NR NR	32	49 7/25	7.1 ± 0.9 NR NR	5.37	55
Jurcut C, 2010, Romania [29]	P	20	62 NR	9.6 ± 0.3 NR 11.8 ± 1.2	64	56 NR	9.0 ± 0.3 NR 14.8 ± 0.9	NR	NR
Yazici S, 2010, Turkey [30]	P	33	48 9/24	8.7 ± 1.3 NR NR	97	51 19/78	9.5 ± 1.3 NR NR	13.9	50
Işık M, 2014, Turkey [31]	R	40	54 12/28	9.2 ± 1.1 NR 16.6 ± 0.4	120	54 39/81	8.4 ± 1.0 NR 16.7 ± 0.7	13	35
Yildirim A, 2015, Turkey [32]	P	52	46 13/39	9.0 ± 2.0 NR 14.0 ± 3.8	90	47 21/69	9.8 ± 0.9 NR 11.4 ± 1.8	12.9	29
Cakır L, 2016, Turkey [33]	R	80	58 27/53	10.3 ± 1.6 13.2 ± 0.8 NR	91	53 17/64	8.9 ± 1.5 14.4 ± 1.0 NR	NR	NR
Gökmen F, 2016, Turkey [34]	P	60	51 16/44	8.9 ± 0.9 NR NR	84	55 20/64	8.5 ± 1.1 NR NR	1.5	32
Tecer D, 2016, Turkey [35]	R	100	58 5/95	9.8 ± 0.5 13.4 ± 0.8 NR	100	58 5/95	10.5 ± 0.9 15.3 ± 2.0 NR	10.5	26
Du J, 2017, China [36]	R	131	43 47/84	NR 12.7 ± 0.6 NR	66	56 14/52	NR 14.1 ± 1.4 NR	10.1	35
Talukdar M, 2017, India [37]	P	80	NR NR	9.40 ± 1.39 NR NR	80	NR 20/60	11.20 ± 1.11 NR NR	NR	NR
Al-Rawi ZS, 2018, Iraq	P	97	48 21/76	NR 12.4 ± 1.1 NR	111	47 13/98	NR 14.5 ± 2.8 NR	NR	NR
Illeez OG, 2018, Turkey [39]	R	104	49 31/73	7.9 ± 1.1 NR 17.6 ± 1.0	105	53 19/86	8.2 ± 1.3 NR 17.5 ± 0.9	0.8	31
Lin F, 2018, China [40]	R	126	45 15/111	NR 13.8 ± 0.8 NR	222	59 44/178	NR 15.1 ± 2.3 NR	NR	NR
Sag S, 2018, Turkey [41]	P	34	50 10/24	8.9 ± 1.3 NR 18.1 ± 1.9	57	53 11/46	7.7 ± 1.5 NR 17.8 ± 1.7	NR	NR
Yang WM, 2018, China [42]	R	159	54 51/108	11.1 ± 1.6 13.0 ± 0.8 NR	160	53 49/111	11.2 ± 1.1 13.6 ± 1.3 NR	21.3	50
Khodashahi M, 2019, Iran [43]	P	35	51 4/31	8.1 ± 1.1 NR NR	105	50 11/94	8.7 ± 1.8 NR NR	6.1	19
Koca TT, 2019, Turkey [44]	R	55	48 12/43	NR 13.4 ± 1.4 NR	120	51 22/98	NR 14.8 ± 2.4 NR	1.2	32
Yildirim OT, 2019, Turkey [45]	P	61	52 NR	10.4 ± 0.9 40.6 ± 5.8 * 12.3 ± 2.0	65	55 NR	10.5 ± 1.0 45.4 ± 5.9 * 12.4 ± 2.4	NR	NR
Khaled SA, 2020, Egypt [46]	P	53	49 NR	8.7 ± 0.9 NR 13.0 ± 2.6	98	49 NR	8.4 ± 1.1 NR 31.7 ± 18.8	18.2	52
Balbaloglu O, 2020, Turkey [47]	P	42	42 NR	7.9 ± 0.9 NR NR	41	46 NR	6.8 ± 1.2 NR NR	10.6	19
Dervisevic A, 2021, Bosnia and Erzegovina [48]	P	34	51 5/29	NR 14.4 ± 0.8 NR	67	55 4/63	NR 15.7 ± 1.7 NR	5.6	NR
Tan L, 2021, China [49]	P	40	51 9/31	NR 13.3 ± 1.6 NR	119	51 29/85	NR 14.6 ± 1.4 NR	NR	NR
Taha SI, 2022, Egypt [50]	P	100	73 49/51	8.4 ± 1.0 13.5 ± 1.6 NR	100	67 19/81	9.8 ± 1.6 15.7 ± 2.4 NR	NR	NR

Legend: NR, not reported; M, male; F, female; P, prospective; R, retrospective; MPV, mean platelet volume; PDW, platelet distribution width; RDW, red cell distribution width; *, fL; CRP, C-reactive protein; ESR, erythrocyte sedimentation rate.

**Table 2:** The Joanna Briggs Institute critical appraisal checklist.

Study	Were the Criteria for Inclusion Clearly Defined?	Were the Subjects and Setting Described in Detail?	Was the Exposure Measured in a Valid Way?	Were Standard Criteria Used to Measure the Condition?	Were Confounding Factors Identified?	Were Strategies to Deal with Confounding Factors Stated?	Were the Outcomes Measured in a Valid Way?	Was Appropriate Statistical Analysis Used?	Risk of Bias
Kisacik [28]	Yes	Yes	Yes	Yes	No	No	Yes	Yes	High
Jurcut [29]	Yes	Yes	Yes	Yes	No	No	Yes	Yes	Low
Yazici [30]	Yes	Yes	Yes	Yes	No	No	Yes	Yes	Low
Işık [31]	Yes	Yes	Yes	Yes	No	No	Yes	Yes	Low
Yildirim [32]	Yes	Yes	Yes	Yes	No	No	Yes	Yes	Low
Cakır [33]	Yes	Yes	Yes	Yes	No	No	Yes	Yes	Low
Gökmen [34]	Yes	Yes	Yes	Yes	No	No	Yes	Yes	Low
Tecer [35]	Yes	Yes	Yes	Yes	No	No	Yes	Yes	Low
Du [36]	Yes	Yes	Yes	Yes	Yes	Yes	Yes	Yes	Low
Talukdar [37]	Yes	Yes	Yes	Yes	No	No	Yes	Yes	Low
Al-Rawi [38]	Yes	Yes	Yes	Yes	No	No	Yes	Yes	Low
Illeez [39]	Yes	Yes	Yes	Yes	No	No	Yes	Yes	Low
Lin [40]	Yes	Yes	Yes	Yes	No	No	Yes	Yes	Low
Sag [41]	Yes	Yes	Yes	Yes	No	No	Yes	Yes	Low
Yang [42]	Yes	Yes	Yes	Yes	Yes	Yes	Yes	Yes	Low
Khodashahi [43]	Yes	Yes	Yes	Yes	No	No	Yes	Yes	Low
Koca [44]	Yes	Yes	Yes	Yes	No	No	Yes	Yes	Low
Yildirim [45]	Yes	Yes	Yes	Yes	No	No	Yes	Yes	Low
Khaled [46]	Yes	Yes	Yes	Yes	No	No	Yes	Yes	Low
Balbaloglu [47]	Yes	Yes	Yes	Yes	No	No	Yes	Yes	Low
Dervisevic [48]	Yes	Yes	Yes	Yes	No	No	Yes	Yes	Low
Tan [49]	Yes	Yes	Yes	Yes	No	No	Yes	Yes	Low
Taha [50]	Yes	Yes	Yes	Yes	No	No	Yes	Yes	Low

## Data Availability

The data that support the findings of this systematic review and meta-analysis are available from the corresponding author, A.Z., upon reasonable request.

## Supplementary Materials

The following are available online at https://www.mdpi.com/article/10.3390/diagnostics12112633/s1, Table S1: PRISMA 2020 abstract checklist; Table S2: PRISMA 2020 manuscript checklist.
